# Value of functional iron parameters in diagnostic re-assessment of MPN: refinement of iron-deficiency markers by zinc protoporphyrin (ZPP)

**DOI:** 10.1007/s00277-026-07077-y

**Published:** 2026-05-23

**Authors:** Kirsi Manz, Myriam Kobrosly, Carl C. Crodel, Haifa Al-Ali, Susanne Isfort, Diana Kleppe, Manuela Neubert, Tina M. Schnöder, Ina Rogalski, Stefanie Jilg, Thomas Stauch, Wolfgang Hoffmann, William H. Krüger, Jan Krönke, Andreas Hochhaus, Christian Homann, Florian H. Heidel

**Affiliations:** 1 Hematology, Hemostasis, Oncology and Cell Therapy, Hannover Medical School (MHH), Hannover, Germany; 2 Institute for Community Medicine, University Medicine Greifswald, Greifswald, Germany; 3 Internal Medicine C, Hematology, Oncology, Stem Cell Transplantation and Palliative Care, University Medicine Greifswald, Greifswald, Germany; 4 Internal Medicine 2, Hematology/Oncology, Jena University Hospital, Jena, Germany; 5 Comprehensive Cancer Center Central Germany – Campus Jena, Jena, Germany; 6 University Hospital Halle and Krukenberg Cancer Center, Halle (Saale), Germany; 7 Onkologie Erding, Erding, Germany; 8 Department of Medicine III, Klinikum rechts der Isar, Technische Universität München, Munich, Germany; 9 Laser Research Laboratory, LIFE Center, University Hospital, LMU Munich, Munich, Germany; 10 FerroSens GmbH, München, Germany

**Keywords:** Iron deficiency, MPN, Fluorescence spectroscopy, Zinc protoporphyrin, ZPP

## Abstract

Iron deficiency assessment in myeloproliferative neoplasms is complicated by chronic inflammation, disease-related anemia, and treatment-induced changes in iron handling. We retrospectively analyzed 445 patients with MPN, including 158 with polycythemia vera, 97 with essential thrombocythemia, 44 with prefibrotic primary myelofibrosis, 127 with overt myelofibrosis, and 19 with MPN-unclassifiable, and compared conventional iron parameters with a population-based SHIP cohort of 4420 participants. ZPP was measured in the MPN cohort using quantitative fluorescence spectroscopy. Compared with SHIP, MPN patients had lower hemoglobin and higher ferritin, and the physiological positive correlation between ferritin and hemoglobin observed in SHIP was reversed in MPN patients, with *R* = 0.27, 95% CI 0.24 to 0.30, *p* < 0.001 in SHIP versus *R* = − 0.45, 95% CI − 0.52 to − 0.36, *p* < 0.001 in MPN. Median ZPP in the MPN cohort was 43.0 µmol/mol heme, with 123 of 445 patients (27.6%) showing ZPP values > 65 µmol/mol heme and 59 of 445 patients (13.3%) showing values > 100 µmol/mol heme. ZPP differed significantly between MPN entities, with median values of 35.0 µmol/mol heme in ET, 49.4 µmol/mol heme in PV, 36.5 µmol/mol heme in pre-PMF, 54.7 µmol/mol heme in overt MF, and 46.0 µmol/mol heme in MPN-U (*p* < 0.001). ZPP correlated inversely with hemoglobin in MPN patients (*R* = − 0.26, 95% CI − 0.35 to − 0.16, *p* < 0.001), whereas its association with ferritin was weak (*R* = − 0.10, 95% CI − 0.20 to − 0.00, *p* = 0.045). Among 80 patients with CTCAE grade ≥ 2 anemia, 58 (73%) had ZPP values above the upper limit of normal. In exploratory multivariable analysis, PV, overt MF, and phlebotomy were independently associated with higher ZPP, whereas CRP and time from diagnosis were not. In JAK2-mutated PV versus ET, ZPP was significantly higher in PV (48.5 versus 37.5 µmol/mol heme, *p* = 0.010), complementing lower transferrin saturation and higher JAK2 variant allele frequency. These data indicate that ZPP provides a functional readout of iron-restricted heme synthesis in MPN, adds information beyond ferritin and transferrin saturation, and may refine diagnostic reassessment and therapeutic monitoring, particularly in PV and overt MF.

## Introduction

Reliable assessment of iron deficiency (ID) is critical in myeloproliferative neoplasms (MPN), particularly when clinicians are confronted with borderline or discordant constellations of hemoglobin (Hb), hematocrit (Hct), and erythropoietin (EPO) values that blur the distinction between polycythemia vera (PV), essential thrombocythemia (ET), prefibrotic primary myelofibrosis (pre‑PMF), overt myelofibrosis (MF), and MPN‑unclassifiable (MPN‑U). In such scenarios, subtle differences in iron metabolism and erythropoietic drive may provide crucial diagnostic cues for reassessment of the underlying MPN subtype. While Hb and Hct remain central to current diagnostic criteria, they largely reflect the net result of clonal erythropoiesis and iron availability [1] and are therefore late and sometimes misleading indicators of functional ID, especially when iron restriction and marrow fibrosis coexist. Mean corpuscular volume (MCV) likewise offers only an indirect readout of iron‑restricted erythropoiesis and can be confounded by coexisting macrocytosis due to vitamin B12 or folate deficiency, cytotoxic therapy, or disease‑related dyserythropoiesis. Consequently, there is a growing need for functional iron markers that can refine diagnostic algorithms in borderline PV‑like or ET‑like cases and in patients with suspected pre‑PMF [2], overt MF, or MPN‑U.

Ferritin, an intracellular iron storage protein widely used as a surrogate of body iron stores, is strongly regulated by inflammatory signaling and therefore poorly suited to the chronic inflammatory milieu that typifies MPN [3]. Persistent activation of JAK–STAT signaling and a complex pro‑inflammatory cytokine network in PV, ET, pre‑PMF, overt MF, and MPN‑U drive an acute‑phase response [4] in which ferritin may be elevated or inappropriately normal despite profound functional iron depletion. Under these conditions, ferritin can no longer be regarded as a bona fide, stand‑alone marker of iron status, and reliance on ferritin risks misclassification of iron‑replete versus iron‑deficient states in diagnostic grey zones. Recent work has therefore shifted attention toward functional iron parameters, showing that transferrin saturation index (TSI), when combined with EPO and JAK2 variant allele frequency (VAF), can improve discrimination between PV and ET in cases with borderline Hb/Hct and overlapping clinical features [5]. This functional approach conceptually supports the use of iron metabolism as a modifier of MPN phenotype but still leaves important gaps in precision, particularly in pre‑PMF, overt MF, and MPN‑U, where inflammation, fibrosis, and ineffective erythropoiesis coexist.

The soluble transferrin receptor (sTFR) and TSI exemplify attempts to move beyond static storage markers. sTFR reflects cellular iron demand and erythropoietic activity and is relatively insensitive to inflammation, whereas TSI integrates circulating iron and transferrin binding capacity to approximate iron immediately available for erythropoiesis. Both parameters have proven useful in general hematology to distinguish absolute from functional ID, but their performance in MPN is modulated by clonal myeloproliferation, altered erythroid kinetics, and disease‑specific therapies such as phlebotomy, cytoreductive agents, and JAK inhibitors. Moreover, neither sTFR nor TSI directly interrogates iron incorporation at the level of heme synthesis, and both can be influenced by nutritional status, iron supplementation, and timing of sampling. Taken together, these limitations underscore the unmet need for an inflammation‑independent, mechanistically proximal biomarker that reflects iron‑restricted heme synthesis and can be integrated with functional iron parameters and JAK2 VAF to support diagnostic reassessment across the MPN spectrum.

The zinc protoporphyrin/heme ratio (in the following abbreviated “ZPP”) fulfils many of these criteria by directly capturing iron availability at the point of heme formation. ZPP is generated when protoporphyrin IX, a key intermediate in the heme biosynthetic pathway, incorporates zinc instead of iron in the setting of insufficient bioavailable iron or impaired iron utilization. Elevated ZPP thus signals iron‑restricted heme synthesis at the erythroid level, independent of ferritin dynamics and largely uncoupled from cytokine‑driven acute‑phase regulation. Clinically, ZPP levels below 40 µmol/mol heme are generally considered normal, 40–65 µmol/mol indicate mild iron deficiency, levels above 65 µmol/mol suggest overt iron deficiency, and values exceeding 100 µmol/mol point to severe functional iron restriction. Earlier work [6–8] established ZPP as a robust tool for staging iron deficiency and differentiating iron deficiency anemia from anemia of chronic disease, and subsequent studies [9] demonstrated that ZPP can be usefully combined with sTFR to dissect mixed etiologies of anemia and to differentiate between PMF and ET [10]. These characteristics make ZPP particularly attractive for MPN, where chronic inflammation, phlebotomy, and clonal erythrocytosis frequently coexist and where a precise readout of functional iron incorporation could sharpen the distinction between iron‑restricted PV‑like states and other MPN subentities.

Historically, the adoption of ZPP in routine hematology has been limited by technical and logistical constraints, including the need for specialized devices, time‑consuming analytical procedures, and the absence of standardized, high‑throughput platforms [11]. Recent advances in quantitative fluorescence spectroscopy now overcome many of these barriers, allowing rapid, sensitive, and low‑cost ZPP measurements on small blood samples and, in some implementations, via non‑invasive mucosal readings [12]. These innovations open the possibility of embedding ZPP into everyday diagnostic workflows for MPN patients, including those undergoing serial phlebotomies, cytoreductive therapy, or JAK inhibition. In the specific context of diagnostic reassessment, ZPP could help identify functionally iron‑deficient, PV‑like disease in patients whose Hb/Hct fall just below formal thresholds, distinguish ET with iron‑restricted erythropoiesis from early PV, and provide an additional layer of information in pre‑PMF, overt MF, and MPN‑U, where conventional iron parameters are difficult to interpret due to inflammation and marrow fibrosis.

The objective of the present study is therefore to establish ZPP measurement using a quantitative fluorescence spectroscopy‑based method in a broad MPN cohort encompassing PV, ET, pre‑PMF, overt MF, and MPN‑U, and to evaluate its role as an inflammation‑independent, functional iron biomarker for diagnostic reassessment in borderline cases. Specifically, we aim to (i) characterize ZPP levels across WHO‑defined MPN subtypes, (ii) compare the diagnostic performance of ZPP for detecting iron depletion with that of Hb, MCV, ferritin, sTFR, TSI, and EPO, and (iii) investigate whether integrating ZPP with functional iron parameters and JAK2 VAF improves discrimination between PV‑like and ET‑like phenotypes and refines classification in patients with suspected pre‑PMF, overt MF, or MPN‑U. By aligning conceptually with previous work that used ferritin and TSI to support diagnostic reassessment, yet replacing ferritin with a mechanistically proximal, inflammation‑independent marker, our study seeks to provide a more precise framework for iron‑based diagnostic refinement across the MPN continuum.

We hypothesize that, in MPN, conventional storage and circulating iron parameters incompletely reflect erythroid iron availability because they are influenced by inflammation, disease stage, and treatment exposure. In this framework, the population-based SHIP cohort serves as a comparator for the physiological relationship between ferritin and hemoglobin in individuals without MPN, whereas ZPP is evaluated exclusively within the MPN cohort as a mechanistically proximal readout of iron-restricted heme synthesis. We therefore aimed to determine whether ZPP provides additional information beyond ferritin, transferrin saturation, soluble transferrin receptor, hemoglobin, erythropoietin, inflammatory markers, treatment exposure, and time from diagnosis, and whether it can refine the characterization of functional iron restriction during diagnostic reassessment across PV, ET, pre-PMF, overt MF, and MPN-U.

## Methods

### Study cohorts

To benchmark ID-markers in a control cohort of non-MPN patients, we retrieved data of the Study of Health in Pomerania (SHIP) conducted at Greifswald University Medicine. SHIP is a population-based epidemiological study consisting of currently 3 independent cohorts [13]. It investigates common risk factors, subclinical disorders and manifest diseases with highly innovative non-invasive methods in the population of northeast Germany. As this study is not focused on one specific disease it aims to investigate health in all aspects and complexity involving the collection and assessment of data pertinent to the prevalence and incidence of common, population-relevant diseases and their risk factors [14]. Data from the SHIP-TREND-0 cohort, collected between September 2008 and September 2012, were used for this analysis. Samples from MPN patients (and healthy donor (HD) controls) were obtained from 5 German centers following approval of the local ethics committees. Primary MPN patient samples and healthy donor controls were obtained after informed consent according to the Helsinki declaration and from the Hematology Tumor Banks Jena, Greifswald and Hannover, approved by the respective local ethics committee (Ethics Committees: University Hospital Jena #4753/04–16; University Medicine Greifswald BB_199 − 20; Hannover Medical School 11501_BO_K_2024).

### ZPP analysis

ZPP measurements were centrally performed between September 2020 and January 2026 using a novel fluorescence spectroscopy-based prototype device (FID*screen*, FerroSens GmbH, München, Germany). For ZPP measurement, 200 µl of peripheral blood (EDTA) was used for the measurement in FID*screen*. Each measurement was performed in technical duplicates per sample to exclude artefacts, and their mean value given as result.

### Genetic analysis

Genomic profiling was performed using the Genexus™ Integrated Sequencer (Thermo Fisher Scientific), which enables automated library preparation, templating and sequencing. Targeted next-generation sequencing was conducted using the Oncomine™ Precision Assay (covering > 30 cancer-related genes [15]), with a mean sequencing depth of > 1,000× and a minimum coverage threshold of 500× for variant calling. Sequence alignment to the human reference genome (hg19) and variant calling were performed using the Torrent Suite™ and Ion Reporter™ software pipelines, applying default quality filters and annotation against curated genomic databases.

### Statistical methods

Standard descriptive statistics were used. Correlations between continuous variables were assessed using Pearson’s correlation coefficient (R). Associations between categorical variables were analyzed using the Fisher’s exact test. Comparisons between two groups were performed using the Mann-Whitney U test, and comparisons across more than two groups using the Kruskal-Wallis test. Exploratory multivariable linear regression was performed using log-transformed ZPP as the dependent variable to assess associations with MPN subtype, age, sex, CRP, treatment exposure, and time from diagnosis. Treatment exposure was evaluated by grouping the therapies into pharmacologic treatment, phlebotomy, watch and wait, and other categories. A p-value < 0.05 was considered statistically significant without adjustment for multiple comparisons. All available MPN data were used, resulting in varying sample sizes across analyses. Data were analyzed and visualized using R (version 4.5.2) [16].

## Results

A total of 445 MPN patients were included in the study (158 (36%) with PV, 97 (22%) with ET, 44 (10%) with pre-PMF, 127 (28%) with overt MF, 19 (4%) with MPN-U, Table 1). The SHIP “general population sample” consisted of 4420 participants. The sex ratio of MPN patients and SHIP participants was similar, but SHIP participants were younger than MPN patients. Overall, Hb was lower and ferritin higher in MPN patients compared to the general population. Transferrin values were comparable.

**Table 1:** Characteristics of MPN and SHIP cohorts

Characteristic	MPN overall (*N* = 445)	SHIP-TREND-0 (*N* = 4420)
Female sex	228 (52.7%)	2275 (51.5%)
Age (years)	60.5 (14.6)	52.0 (15.5) ***
Hb (g/dL) (missing *n* = 66/15)	12.2 (2.5)	14.0 (1.2)***
Ferritin (µg/l) (missing *n* = 56/156)	308 (658)	131 (153) ***
Transferrin (mg/ml) (missing *n* = 344/158)	2.5 (0.4)	2.6 (0.4) *
ZPP (µmol/mol heme) Median [Range]	43.0 [13.0, 418.0]	
ZPP > 65	*n* = 123 (27.6%)	
ZPP > 100	*n* = 59 (13.3%)	
Time between diagnosis and ZPP measurement (years) Median [Range]	4.2 [0.0, 37.1]	

Continuous variables are summarized as mean (standard deviation), except ZPP and time between diagnosis and ZPP measurement, which are summarized as median [range]. Missing numbers indicate the number of missing values in the overall MPN cohort / the SHIP cohort, where applicable. Hb = hemoglobin, ZPP = zinc protoporphyrin. Statistically significant differences between MPN patients and SHIP participants are shown as * (*p* < 0.05), ** (*p* < 0.01) or *** (*p* < 0.001) in the rightmost column. Percentages are calculated among non-missing observations

**Fig. 1:**
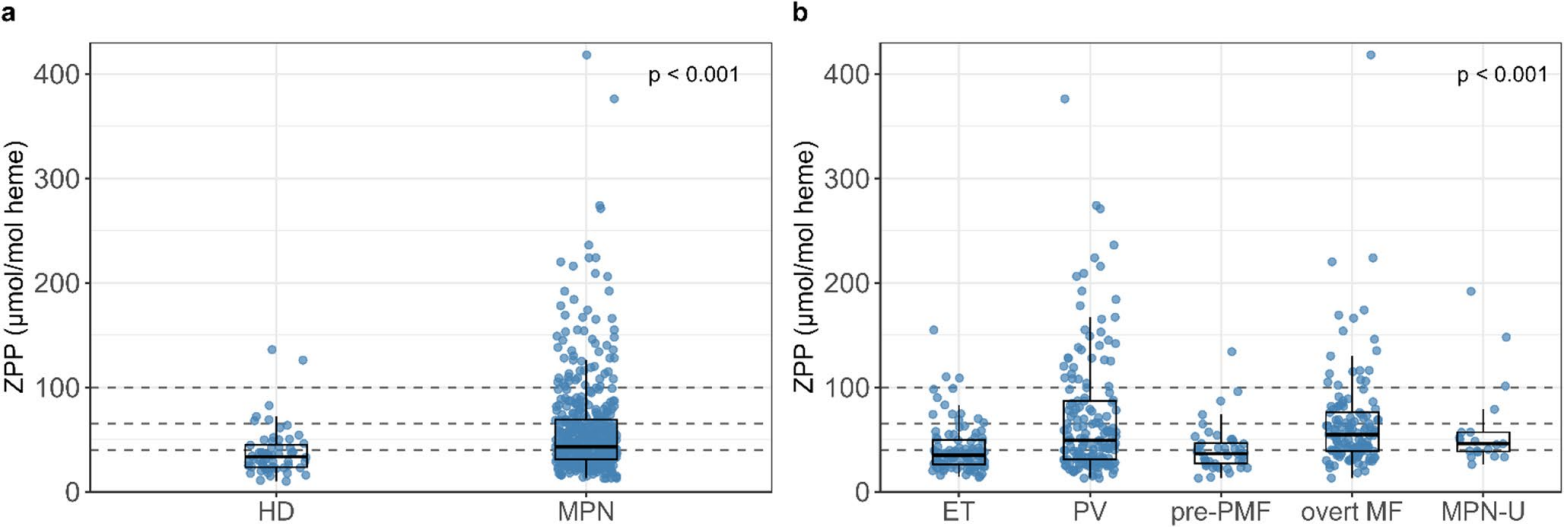
Zinc protoporphyrin (ZPP) in healthy donors (HD; *n* = 60) and patients with myeloproliferative neoplasms (MPN *n* = 445) (**a**), ZPP in patients essential thrombocythemia (ET *n* = 97), with polycythemia vera (PV *n* = 158), prefibrotic primary myelofibrosis (pre-PMF *n* = 44), overt myelofibrosis (MF *n* = 127) and unclassifiable MPN (MPN-U *n* = 19) (**b**). Gray dashed horizontal lines indicate the ZPP thresholds of 40, 65, and 100 µmol/mol heme

ZPP measurement was performed a median of 4.2 years (range: 0-37.1 years) after diagnosis of MPN. With a median of 34 and 43 µmol/mol heme, ZPP levels were significantly lower in 60 healthy donors compared to MPN patients (Fig. 1a), respectively, indicating a lower rate of ID. ZPP values < 40 µmol/mol heme (ULN), indicating no ID, were recorded in 41/60 (68%) HD, values between 40 and 65 µmol/mol heme in 13/60 (22%) HD, and values > 65 µmol/mol heme, clearly indicating ID, in 6/60 (10%) HD, including 2/60 (3%) > 100 µmol/mol heme. In contrast, 191/445 (43%) of MPN patients showed ZPP levels < 40 µmol/mol heme, while 59/445 (13%) showed values > 100 µmol/mol heme, consistent with severe ID. Of note, ZPP levels differed significantly between MPN entities, and all groups included patients with severe ID (Fig. 1b). Markedly elevated ZPP levels were observed particularly in PV and overt MF patients. Among PV patients, 34/158 (22%) had ZPP levels of > 100 µmol/mol heme, likely reflecting phlebotomy-induced ID, as phlebotomies are standard of care in PV [17, 18]. Data on phlebotomy-dependence were available for 129 patients with PV. In this subgroup, median ZPP values were significantly higher in patients on phlebotomy treatment than in patients without (65 vs. 43 µmol/mol heme, *p* = 0.031).

Exploratory multivariable analysis was performed in 220 patients with complete data after exclusion of 2 patients receiving other therapies (standard of care). Using log-transformed ZPP as the dependent variable, PV, overt MF, and phlebotomy treatment were independently associated with higher ZPP levels (Table 2). Male sex was associated with lower ZPP levels. In contrast, log-transformed CRP and time from diagnosis were not independently associated with ZPP after adjustment for MPN subtype, age, sex, and treatment exposure. Pharmacologic therapy was associated with lower ZPP levels, although this association did not reach statistical significance.

**Table 2:** Multivariable linear regression for log-transformed ZPP

Variable	Beta	95% CI	*p*-value
PV (vs. ET)	0.317	0.114 to 0.521	**0.002**
pre-PMF (vs. ET)	0.079	-0.156 to 0.314	0.509
overt MF (vs. ET)	0.532	0.323 to 0.740	**< 0.001**
MPN-U (vs. ET)	0.259	-0.167 to 0.685	0.231
Age (years)	-0.005	-0.010 to 0.001	0.078
Male sex	-0.152	-0.295 to -0.009	**0.038**
log(CRP)	0.013	-0.046 to 0.072	0.662
Phlebotomy (vs. watch and wait)	0.384	0.077 to 0.692	**0.015**
Pharmacologic therapy (vs. watch and wait)	-0.126	-0.284 to 0.033	0.120
Time from diagnosis (years)	0.007	-0.002 to 0.016	0.139

Reference categories: ET, female sex, watch and wait. CRP (C-reactive protein) was included as log-transformed variable. Beta = regression coefficient, CI = confidence interval

**Fig. 2:**
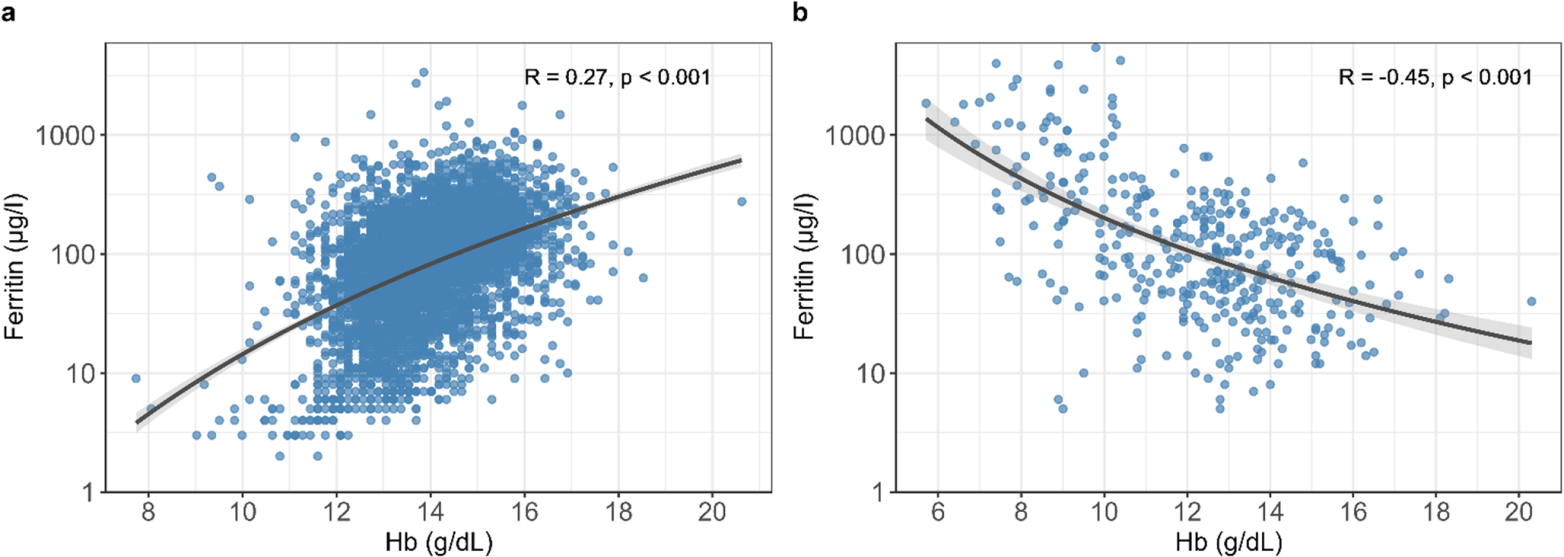
Correlation between ferritin and hemoglobin (Hb) in the general population of the SHIP cohort *n* = 4258 (**a**), correlation between ferritin and Hb in MPN patients *n* = 370 (**b**). Gray areas indicate the confidence interval for the regression line shown as a black line. Ferritin is shown on a logarithmic scale

In the general population there was a positive correlation between Hb and ferritin (*R* = 0.27 (CI: 0.24, 0.30), *p* < 0.001, Fig. 2a). In MPN patients, however, the correlation between Hb and ferritin was negative, with *R* = -0.45 (CI: -0.52, -0.36), *p* < 0.001 (Fig. 2b), thus limiting the use of ferritin as indicator for ID.

**Fig. 3:**
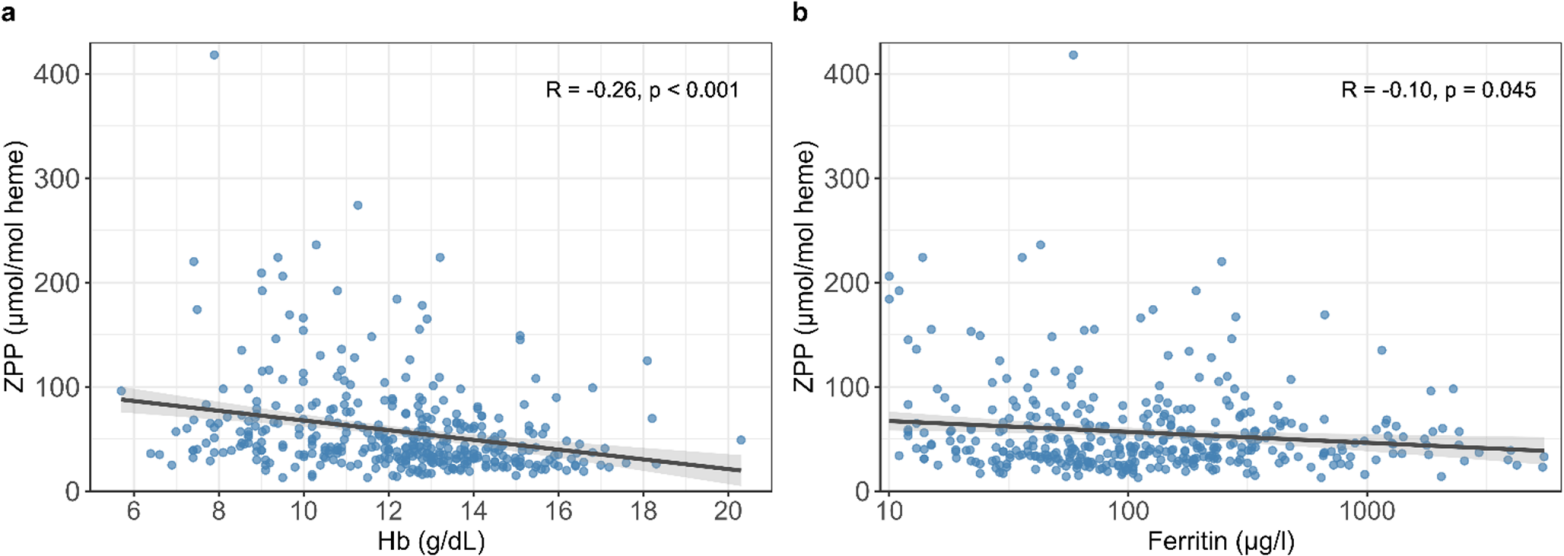
Correlation between zinc protoporphyrin (ZPP) and hemoglobin (Hb) in MPN patients *n* = 379 (**a**), and correlation between ZPP and ferritin in MPN patients *n* = 389 (**b**). Gray areas indicate the confidence interval for the regression line shown as a black line. Ferritin is shown on a logarithmic scale

ZPP and Hb were negatively associated in MPN patients (*R* = -0.26 (CI: -0.35, -0.16), *p* < 0.001, Fig. 3a), whereas ZPP and ferritin showed only a very weak negative association (*R* = -0.10 (CI: -0.20, -0.00), *p* = 0.045, Fig. 3b). Considering the threshold for grade 2 anemia of 10.0 g/dL according to the National Cancer Institute’s Common Terminology Criteria for Adverse Events (CTCAE) grading system [19], the ZPP values of the 80 anemic patients were above the ULN in 58 cases (73%). ZPP and sTFR were positively correlated (*R* = 0.45 (CI: 0.29, 0.59), *p* < 0.001), but sTFR and Hb did not show a correlation (*R* = 0.03 (CI: -0.15, 0.22), *p* = 0.723).

### General characteristics of the cohort

A total of 445 patients with chronic myeloproliferative neoplasms (CMPN) were included, comprising 97 (21.8%) with ET, 158 (35.5%) with PV, 44 (9.9%) with pre-PMF, 127 (28.5) with overt MF, and 19 (4.3%) with MPN‑U (Table 3). The median age at study entry was 62.2 years (IQR 51.9–71.6), with significant differences across entities (p = < 0.001): ET patients were younger (57.4 years, IQR 45.7–66.8) than those with PV (63.5 years, IQR 51.7–72.7), pre-PMF (58.9 years, IQR 49.2–67.9), overt MF (65.2 years, IQR 56.8–73.2), and MPN‑U (68.3 years, IQR 54.8–74.4). Sex distribution did not differ significantly between entities (*p* = 0.189); women represented 60.0% of ET, 55.3% of PV, 45.5% of pre-PMF, 45.7% of overt MF, and 58.8% of MPN‑U cases. Driver mutation analysis was available for 394 patients and revealed a typical MPN pattern with entity‑specific differences (*p* < 0.001). JAK2 mutations were most frequent overall and enriched in PV (97.3%) relative to ET (56.8%), pre-PMF (47.7%), overt MF (54.8%), and MPN‑U (37.5%). CALR mutations were absent in PV but common in ET (26.3%), pre-PMF (38.6%) and overt MF (31.7%), while MPL mutations occurred at low frequency in ET (3.2%), pre-PMF (4.5%) and overt MF (5.6%). Composite driver constellations (e.g. JAK2 + MPL, CALR + MPL, JAK2 + CALR) were rare, and triple‑negative disease was particularly over‑represented in MPN‑U (50.0%) compared with ET (8.4%), overt MF (7.1%), pre-PMF (4.5%) and PV (0.9%). In contrast to the González‑Resina cohort, which was restricted to PV and ET and characterized mainly by ferritin, TSI, and JAK2 VAF, our cohort spans the full CMPN spectrum and systematically integrates ZPP as a direct functional iron marker, allowing assessment of iron‑restricted erythropoiesis beyond storage parameters across all subtypes.

**Table 3:** Mutation status by entity (ET, PV, pre-PMF, overt MF, MPN-U)

Characteristic	*N*	ET *N* = 97	PV *N* = 158	Pre-PMF *N* = 44	Overt MF *N* = 127	MPN-U *N* = 19	*p*-value
Age (years)	443	57.4 (45.7–66.8)	63.5 (51.7–72.7)	58.9 (49.2–67.9)	65.2 (56.8–73.2)	68.3 (54.8–74.4)	**< 0.001**
Sex	433						0.189
female		57 (60.0%)	83 (55.3%)	20 (45.5%)	58 (45.7%)	10 (58.8%)	
male		38 (40.0%)	67 (44.7%)	24 (54.5%)	69 (54.3%)	7 (41.2%)	
Driver mutation	394						**< 0.001**
JAK2		54 (56.8%)	110 (97.3%)	21 (47.7%)	69 (54.8%)	6 (37.5%)	
CALR		25 (26.3%)	0 (0.0%)	17 (38.6%)	40 (31.7%)	2 (12.5%)	
MPL		3 (3.2%)	0 (0.0%)	2 (4.5%)	7 (5.6%)	0 (0.0%)	
JAK2 + MPL		3 (3.2%)	2 (1.8%)	1 (2.3%)	1 (0.8%)	0 (0.0%)	
CALR + MPL		1 (1.1%)	0 (0.0%)	1 (2.3%)	0 (0.0%)	0 (0.0%)	
JAK2 + CALR		1 (1.1%)	0 (0.0%)	0 (0.0%)	0 (0.0%)	0 (0.0%)	
Triple negative		8 (8.4%)	1 (0.9%)	2 (4.5%)	9 (7.1%)	8 (50.0%)	
Ferritin (ng/mL)	389	87.5 (52.5–171.4)	43.0 (19.0–119.0)	127.5 (49.0–276.0)	230.7 (88.0–656.0)	192.5 (69.0–1,271.0)	**< 0.001**
ZPP (µmol/mol heme)	445	35.0 (26.3–49.5)	49.4 (31.0–87.0)	36.5 (27.0–47.0)	54.7 (39.0–76.0)	46.0 (38.0–57.0)	**< 0.001**
TSI (%)	386	26.0 (19.5–30.0)	17.0 (8.0–28.0)	26.0 (19.0–31.0)	30.0 (20.5–43.0)	29.0 (22.0–81.0)	**< 0.001**
EPO (mIU/mL)	262	7.0 (4.3–9.7)	4.1 (2.5–10.9)	16.3 (5.5–48.8)	54.2 (17.9–179.5)	35.3 (9.1–57.9)	**< 0.001**
Hb (g/dL)	379	13.3 (12.5–14.3)	13.6 (12.4–15.1)	12.1 (10.5–13.4)	10.0 (8.9–11.8)	10.5 (8.7–12.3)	**< 0.001**
Hct (%)	280	39.0 (36.5–41.6)	42.5 (37.6–46.1)	35.2 (31.0–40.7)	30.2 (26.2–34.1)	29.3 (24.2–36.8)	**< 0.001**
CRP (mg/L)	240	0.8 (0.6–1.5)	1.1 (0.6–3.2)	1.1 (0.6–4.3)	2.5 (0.6–5.9)	3.0 (0.9–10.4)	**0.006**

Continuous variables are presented as median (interquartile range), and categorical variables as counts (percentages). ZPP = zinc protoporphyrin, TSI = transferrin saturation index, EPO = erythropoietin, Hb = hemoglobin, Hct = hematocrit, CRP = C-reactive protein

### Clinical and biochemical comparison between MPN subtypes

Across entities, iron‑related and erythropoietic parameters showed distinct patterns that reflect both disease biology and stage. Median ferritin levels differed markedly between subtypes (*p* < 0.001), with the lowest values in ET (87.5 ng/mL, IQR 52.5–171.4) and PV (43.0 ng/mL, IQR 19.0–119.0), and substantially higher levels in pre-PMF (127.5 ng/mL, IQR 49.0–276.0), overt MF (230.7 ng/mL, IQR 88.0–656.0) and MPN‑U (192.5 ng/mL, IQR 69.0–1,271.0, Table 3). This pattern is consistent with the González‑Resina PV/ET data, where ferritin was lower in PV than ET, but our broader panel shows that ferritin in advanced or inflammatory entities (MF, MPN‑U) is dominated by acute‑phase and disease‑stage effects and thus poorly reflects functional iron restriction.

In contrast, ZPP values consistently tracked functional iron‑restricted erythropoiesis across subtypes (*p* < 0.001). Median ZPP was lowest in ET (35.0 µmol/mol heme, IQR 26.3–49.5) and pre-PMF (36.5 µmol/mol heme, IQR 27.0–47.0), but significantly higher in PV (49.4 µmol/mol heme, IQR 31.0–87.0), overt MF (54.7 µmol/mol heme, IQR 39.0–76.0), and MPN‑U (46.0 µmol/mol heme, IQR 38.0–57.0). Thus, despite similar or even markedly elevated ferritin in overt MF and MPN‑U, ZPP values fell into the range of overt functional iron deficiency, indicating iron‑restricted heme synthesis that is not apparent from ferritin alone. When viewed in the light of González‑Resina’s finding that TSI discriminates PV from ET better than ferritin, these data show that ZPP adds a mechanistically proximal readout of iron incorporation that remains informative even where ferritin is strongly driven by inflammation and disease progression.

**Table 4:** Iron status parameters by driver mutation

Characteristic	*N*	JAK2 *N* = 260	CALR *N* = 84	MPL *N* = 12	Triple negative *N* = 28	*p*-value
Ferritin (ng/mL)	372	87.8 (33.5–218.5)	107.0 (64.0–308.0)	276.7 (201.0–443.6)	173.0 (63.0–316.0)	**< 0.001**
ZPP (µmol/mol heme)	384	46.6 (31.7–70.4)	39.5 (31.0–62.0)	54.5 (35.0–66.6)	40.0 (31.0–48.0)	0.224
TSI (%)	369	24.0 (15.0–31.0)	28.0 (23.0–34.0)	37.0 (24.0–47.0)	28.0 (19.0–35.0)	**< 0.001**
EPO (mIU/mL)	253	5.6 (3.7–19.3)	20.2 (12.9–62.4)	57.3 (19.4–96.9)	9.1 (5.1–57.9)	**< 0.001**
Hb (g/dL)	365	12.8 (10.8–14.1)	11.9 (10.2–13.0)	9.8 (8.8–11.5)	11.0 (9.2–13.1)	**< 0.001**
Hct (%)	268	39.1 (32.7–43.6)	35.6 (31.0–39.1)	27.6 (26.8–29.4)	32.2 (27.1–38.9)	**< 0.001**
CRP (mg/L)	230	1.1 (0.6–4.2)	0.8 (0.6–2.1)	1.5 (0.6–2.2)	2.9 (1.0–5.9)	**0.016**

Values are presented as median (interquartile range). ZPP = zinc protoporphyrin, TSI = transferrin saturation index, EPO = erythropoietin, Hb = hemoglobin, Hct = hematocrit, CRP = C-reactive protein

TSI exhibited a biphasic pattern across entities (*p* < 0.001). As in González‑Resina, PV patients displayed lower TSI (17.0%, IQR 8.0–28.0) than ET (26.0%, IQR 19.5–30.0), consistent with phlebotomy‑ and erythrocytosis‑related iron restriction. However, overt MF and MPN‑U showed higher TSI values (30.0%, IQR 20.5–43.0 and 29.0%, IQR 22.0–81.0, respectively) despite elevated ferritin, reflecting relative iron re‑distribution and ineffective erythropoiesis rather than absolute deficiency. Pre-PMF showed similar TSI values to ET. Importantly, ZPP remained elevated in these entities, indicating that even with preserved or increased circulating iron (TSI), erythroid iron utilization at the heme level is impaired. This divergence between TSI and ZPP underlines the added diagnostic value of ZPP beyond the functional iron measures evaluated by González‑Resina. EPO and red cell indices further differentiated entities. EPO was lowest in ET (7.0 mIU/mL, IQR 4.3–9.7) and PV (4.1 mIU/mL, IQR 2.5–10.9), and markedly elevated in pre-PMF (16.3 mIU/mL, IQR 5.5–48.8), overt MF (54.2 mIU/mL, IQR 17.9–179.5) and MPN‑U (35.3 mIU/mL, IQR 9.1–57.9; *p* < 0.001). Hb and Hct were largely preserved in ET and PV (Hb 13.3 and 13.6 g/dL; Hct 39.0% and 42.5%, respectively) but significantly reduced in overt MF and MPN‑U (Hb ~ 10.0–10.5 g/dL; Hct ~ 29–30%; both *p* < 0.001). Pre-PMF showed values in-between (Hb 12.1 g/dL; Hct 35.2%). CRP values varied with *p* = 0.006 significantly between the subtypes and were the lowest in ET (0.8 mg/L, IQR 0.6–1.5) and the highest in overt MF and MPN-U (2.5 mg/L, IQR 0.6–5.9 and 3.0 mg/L, IQR 0.9–10.4). In this context, elevated ZPP in MF and MPN‑U identifies iron‑restricted erythropoiesis despite high ferritin and EPO, highlighting functional iron deficiency as a contributor to anemia that would be underestimated with ferritin‑focused assessment alone.

### Analytical analysis according to mutational profile

When stratified by driver mutation, ferritin, TSI, EPO, Hb, and Hct displayed the expected mutation‑specific profiles, while ZPP remained surprisingly homogeneous. Among 384 patients with available ZPP and mutation data, median ZPP did not differ significantly across JAK2 (46.6 µmol/mol heme, IQR 31.7–70.4), CALR (39.5 µmol/mol heme, IQR 31.0–62.0), MPL (54.5 µmol/mol heme, IQR 35.0–66.6), and triple‑negative disease (40.0 µmol/mol heme, IQR 31.0–48.0; *p* = 0.224, Table 4). This indicates that functional iron‑restricted heme synthesis, as captured by ZPP, is a shared feature across molecular subgroups and is driven more by disease entity and stage than by the specific driver mutation alone.

In contrast, ferritin and TSI varied significantly by mutation type. Ferritin was lowest in JAK2‑mutated patients (87.8 ng/mL, IQR 33.5–218.5) and higher in CALR (107.0 ng/mL, IQR 64.0–308.0), MPL (276.7 ng/mL, IQR 201.0–443.6), and triple‑negative disease (173.0 ng/mL, IQR 63.0–316.0; *p* < 0.001). TSI was also lowest in JAK2‑mutated cases (24.0%, IQR 15.0–31.0) compared with CALR (28.0%, IQR 23.0–34.0), MPL (37.0%, IQR 24.0–47.0), and triple‑negative disease (28.0%, IQR 19.0–35.0; *p* < 0.001). These findings conceptually mirror González‑Resina’s observation that JAK2‑mutated CMPN exhibit lower ferritin and TSI than CALR/MPL/negative cases, but our data demonstrate that ZPP captures functional iron restriction across all mutational backgrounds, even when ferritin and TSI vary widely.

EPO and erythroid parameters also differed by mutation. JAK2‑mutated patients had lower EPO (5.6 mIU/mL, IQR 3.7–19.3) than CALR (20.2 mIU/mL, IQR 12.9–62.4), MPL (57.3 mIU/mL, IQR 19.4–96.9), and triple‑negative patients (9.1 mIU/mL, IQR 5.1–57.9; *p* < 0.001). Hb and Hct were highest in JAK2‑mutated cases (12.8 g/dL, IQR 10.8–14.1; 39.1%, IQR 32.7–43.6) and progressively lower in CALR, MPL, and triple‑negative groups (both *p* < 0.001). CRP values also differed by mutation, with lowest values in CALR-mutated patients (0.8 mg/L, IQR 0.6–2.1) and highest values in triple negative patients (2.9 mg/L, IQR 1.0-5.9; *p* = 0.016). Taken together, these results support the González‑Resina conclusion that mutational status and allele burden modulate hematologic phenotype, while extending it by showing that ZPP reflects iron‑restricted erythropoiesis across all mutation classes and therefore provides a mutation‑agnostic measure of functional iron deficiency.

**Table 5:** Iron status in JAK2-positive ET, PV, pre-PMF and overt PMF

Characteristic	*N*	ET *N* = 54	PV *N* = 110	pre-PMF *N* = 21	Overt MF *N* = 69	*p*-value
Ferritin (ng/mL)	246	72.2 (55.0–186.9)	45.5 (22.0–124.2)	114.0 (31.0–381.5)	187.6 (67.0–662.2)	**< 0.001**
ZPP (µmol/mol heme)	254	37.5 (27.8–56.0)	48.5 (31.0–81.0)	34.0 (25.0–50.0)	57.0 (40.0–74.0)	**< 0.001**
TSI (%)	244	26.0 (18.5–30.5)	18.0 (8.0–28.0)	25.5 (18.5–39.0)	27.0 (18.0–40.0)	**< 0.001**
EPO (mIU/mL)	166	5.4 (4.0–7.7)	4.1 (2.5–10.9)	9.8 (4.4–27.3)	26.0 (6.4–180.0)	**< 0.001**
Hb (g/dL)	239	13.4 (12.8–14.5)	13.5 (12.4–15.1)	12.1 (10.4–14.1)	10.2 (8.9–11.9)	**< 0.001**
Hct (%)	174	39.1 (37.0–41.7)	42.5 (37.6–46.1)	35.2 (31.0–41.1)	31.3 (25.8–35.1)	**< 0.001**
CRP (mg/L)	149	0.8 (0.6–1.7)	1.1 (0.6–3.2)	0.9 (0.6–3.7)	4.0 (0.9–8.0)	**0.024**
VAF (%)	234	8.9 (5.0–19.3)	32.5 (18.0–50.4)	17.6 (12.0–42.5)	38.8 (22.4–84.0)	**< 0.001**

Values are presented as median (interquartile range). ZPP = zinc protoporphyrin, TSI = transferrin saturation index, EPO = erythropoietin, Hb = hemoglobin, Hct = hematocrit, CRP = C-reactive protein, VAF = variant allele frequency

## Analytical subanalysis in patients with JAK2‑positive CMPN

We next focused on JAK2‑mutated ET and PV, analogous to the analytical subanalysis performed by González‑Resina, but with the addition of ZPP and an extension into pre-PMF and overt MF. Among JAK2‑positive patients with ET and PV, PV (*n* = 110) had lower ferritin than ET (*n* = 54) (45.5 vs. 72.2 ng/mL; *p* = 0.002) and lower TSI (18.0% vs. 26.0%; *p* = 0.001, Table 5), confirming more pronounced storage and circulating iron depletion in PV within the same mutational background. This pattern parallels González‑Resina’s observation that TSI is more sensitive than ferritin for distinguishing PV from ET in JAK2‑mutated CMPN, but our data show that both parameters can be complemented by a direct functional marker. JAK2-mutated pre-MPF (*n* = 21) and overt MF (*n* = 69) patients had markedly higher ferritin values than patients with ET or PV. TSI values were comparable to ET patients.

ZPP provided this additional, mechanistically proximal separation between JAK2‑positive ET and PV. Median ZPP was significantly higher in PV than in ET (48.5 vs. 37.5 µmol/mol heme; *p* = 0.010), indicating more pronounced iron‑restricted heme synthesis in PV despite overlapping ranges of ferritin and TSI. Of the 4 subtypes, ZPP was with 34.0 µmol/mol heme the lowest in JAK2-positive pre-PMF patients and with 57.0 µmol/mol heme the highest in JAK2-positive overt MF patients. Hb and Hct were broadly comparable between JAK2‑mutated PV and ET (Hb 13.5 vs. 13.4 g/dL, *p* = 0.953; Hct 42.5% vs. 39.1%, *p* = 0.022 with modest effect size), underscoring that ZPP detects functional iron restriction in a range where classical erythroid indices alone do not clearly separate the entities. Hb and Hct were the lowest in JAK2-mutated patients with overt MF (Hb 10.2 g/dL; Hct 31.3%). EPO levels did not differ significantly between JAK2‑mutated ET and PV (5.4 vs. 4.1 mIU/mL; *p* = 0.154). However, EPO was with 26.0 mIU/mL the highest in JAK2-positive patients with overt MF, similarly to CRP (4.0 mg/L)). JAK2 VAF was substantially higher in overt MF and PV compared to ET (38.8% and 32.5% vs. 8.9%; *p* < 0.001), supporting the concept that higher allelic burden and higher ZPP jointly delineate a phenotype of clonal erythrocytosis with iron‑restricted heme synthesis.

**Table 6:** Iron parameters in ET: JAK2-positive vs. No JAK2

Characteristic	*N*	JAK2-positive *N* = 54	No JAK2 *N* = 36	*p*-value
Ferritin (ng/mL)	88	72.2 (55.0–186.9)	91.0 (52.5–132.0)	0.908
ZPP (µmol/mol heme)	90	37.5 (27.8–56.0)	34.5 (24.5–41.5)	0.231
TSI (%)	87	26.0 (18.5–30.5)	26.0 (21.0–31.0)	0.900
EPO (mIU/mL)	56	5.4 (4.0–7.7)	9.0 (6.5–14.5)	**0.004**
Hb (g/dL)	89	13.4 (12.8–14.5)	12.9 (12.0–14.2)	0.143
Hct (%)	61	39.1 (37.0–41.7)	39.2 (36.7–41.5)	0.811
CRP (mg/L)	55	0.8 (0.6–1.7)	0.8 (0.6–1.1)	0.428

Values are presented as median (interquartile range). ZPP = zinc protoporphyrin, TSI = transferrin saturation index, EPO = erythropoietin, Hb = hemoglobin, Hct = hematocrit, CRP = C-reactive protein

### Comparative analysis in patients with essential thrombocythemia according to JAK2 mutation status

Within the ET subgroup, we compared laboratory parameters between JAK2‑mutated (*n* = 54) and non‑JAK2 ET (CALR/MPL/triple‑negative, *n* = 36). In contrast to González‑Resina, where JAK2‑mutated ET showed significantly lower ferritin and TSI than non‑JAK2 ET, ferritin and TSI did not differ in our cohort: median ferritin was 72.2 ng/mL in JAK2‑positive ET versus 91.0 ng/mL in non‑JAK2 ET (*p* = 0.908), and TSI was 26.0% in both groups (*p* = 0.900, Table 6). These findings suggest that, in real‑world ET populations, storage and circulating iron parameters alone may be insufficiently sensitive to capture mutationally driven differences in iron handling, especially under variable treatment and inflammatory conditions. ZPP again provided additional granularity. Although median ZPP tended to be higher in JAK2‑mutated ET than in non‑JAK2 ET (37.5 vs. 34.5 µmol/mol heme), this difference did not reach statistical significance (*p* = 0.231), but both distributions lay in the range compatible with mild functional iron deficiency. EPO levels, by contrast, were significantly lower in JAK2‑positive ET (5.4 mIU/mL) than in non‑JAK2 ET (9.0 mIU/mL; *p* = 0.004), consistent with stronger clonal erythropoietic drive and relative EPO suppression in JAK2‑mutated disease. Hb and Hct were comparable between JAK2‑positive and non‑JAK2 ET (Hb 13.4 vs. 12.9 g/dL, *p* = 0.143; Hct 39.1% vs. 39.2%, *p* = 0.811), indicating that differences in iron utilization and EPO response do not necessarily translate into overt differences in red cell indices within ET. Also CRP levels did not differ between JAK2-positive and non-JAK2 ET (both 0.8 mg/L, *p* = 0.428). In this setting, ZPP offers a stable, inflammation‑independent measure of functional iron status that can be integrated with EPO and mutation profile for diagnostic reassessment in borderline ET‑like cases, complementing the TSI‑ and ferritin‑based approach described by González‑Resina.

### Comparative analysis in patients with prefibrotic primary myelofibrosis and overt myelofibrosis according to JAK2 mutation status

In pre-PMF, iron parameters were broadly comparable between JAK2-positive (*n* = 21) and non-JAK2 (*n* = 21) patients (Table 7). Ferritin, ZPP, Hb, Hct, and CRP did not differ significantly between groups, while EPO levels tended to be lower in JAK2-positive pre-PMF (9.8 vs. 19.4 mIU/mL; *p* = 0.062). ZPP levels in pre-PMF were similar in JAK2-positive and non-JAK2 disease (34.0 vs. 36.0 µmol/mol heme; *p* = 0.860).

In overt MF, substantially higher ZPP levels were observed in both JAK2-positive (*n* = 69) and non-JAK2 (*n* = 56) patients compared with pre-PMF, indicating pronounced iron-restricted erythropoiesis irrespective of driver mutation status. Ferritin levels tended to be lower in JAK2-positive overt MF (187.6 vs. 246.4 ng/mL; *p* = 0.280), whereas TSI was significantly lower in JAK2-positive overt MF than in non-JAK2 overt MF (27.0% vs. 32.5%; *p* = 0.019). Hb and Hct were comparably reduced in both overt MF groups, and no statistically significant differences in ZPP or EPO levels were observed according to mutation status. CRP levels likewise tended to be higher in overt MF than in pre-PMF, although no statistically significant differences according to mutation status were observed within the overt MF subgroup.

**Table 7:** Iron parameters in pre-PMF and overt MF: JAK2-positive vs. No JAK2

Characteristic	pre-PMF				Overt MF			
	*N*	JAK2-positive *N* = 21	No JAK2 *N* = 21	*p*-value	*N*	JAK2-positive *N* = 69	No JAK2 *N* = 56	*p*-value
Ferritin (ng/mL)	41	114.0 (31.0–381.5)	137.0 (82.0–276.0)	0.434	120	187.6 (67.0–662.2)	246.4 (95.0–643.0)	0.280
ZPP (µmol/mol heme)	42	34.0 (25.0–50.0)	36.0 (27.0–44.0)	0.860	125	57.0 (40.0–74.0)	52.0 (39.0–80.3)	0.745
TSI (%)	41	25.5 (18.5–39.0)	27.0 (19.0–31.0)	0.948	118	27.0 (18.0–40.0)	32.5 (26.0–45.5)	**0.019**
EPO (mIU/mL)	41	9.8 (4.4–27.3)	19.4 (14.9–54.3)	0.062	68	26.0 (6.4–180.0)	59.9 (21.2–175.5)	0.156
Hb (g/dL)	41	12.1 (10.4–14.1)	12.1 (10.6–13.2)	0.865	119	10.2 (8.9–11.9)	9.9 (8.9–11.8)	0.823
Hct (%)	42	35.2 (31.0–41.1)	35.2 (30.9–38.7)	0.910	76	31.3 (25.8–35.1)	29.6 (26.9–33.8)	0.650
CRP (mg/L)	35	0.9 (0.6–3.7)	1.5 (0.6–6.3)	0.346	61	4.0 (0.9–8.0)	1.5 (0.6–3.9)	0.092

Values are presented as median (interquartile range). ZPP = zinc protoporphyrin, TSI = transferrin saturation index, EPO = erythropoietin, Hb = hemoglobin, Hct = hematocrit

Taken together, our findings show that across the MPN spectrum ferritin and TSI are strongly modulated by disease entity, inflammation, and mutational context, whereas ZPP consistently reflects iron‑restricted heme synthesis at the erythroid level. In PV and JAK2‑mutated CMPN, ZPP parallels and refines the functional iron restriction signature inferred from low TSI and high JAK2 VAF as described by González‑Resina, but additionally identifies patients with preserved or even elevated ferritin who nonetheless exhibit clear functional iron deficiency. In MF and MPN‑U, ZPP uncovers a substantial subset of patients with “normal” or increased ferritin and near‑normal TSI who are functionally iron deficient, a pattern that would remain largely unrecognized using storage and circulating iron parameters alone. These observations suggest that integrating ZPP into diagnostic reassessment algorithms has the potential to reclassify clinically relevant subgroups as iron‑restricted despite apparently reassuring ferritin/TSI profiles, thereby providing a mechanistically grounded framework for interpreting iron status in MPN and motivating a re‑evaluation of ferritin‑centric approaches in this inflammatory disease context.

## Discussion

Using a novel and applicable method for clinical routine measurement, ZPP was confirmed in our study as a robust marker of iron deficiency (ID) in MPN that overcomes key limitations of conventional iron parameters. In the general population, ferritin usually reflects iron stores and correlates positively with hemoglobin (Hb), but in MPN this relationship was reversed, with higher ferritin at lower Hb, particularly in myelofibrosis (MF), where anemia was most pronounced despite the highest ferritin levels. This pattern is compatible with inflammation-driven hyperferritinemia and iron sequestration and illustrates that ferritin no longer reliably indicates usable iron in MPN. Across the MPN spectrum, there was a consistent dissociation between storage iron and functional iron incorporation into heme. Ferritin and transferrin saturation index (TSI) varied by entity, mutation, and inflammatory burden, whereas ZPP showed a stable pattern of iron-restricted heme synthesis.

Importantly, beyond the general concept of iron deficiency (ID), our findings also highlight a clinically highly relevant aspect that is not sufficiently captured by conventional terminology: the detection and quantification of iron-deficient erythropoiesis prior to the development of overt ID. This is particularly relevant in patients with polycythemia vera (PV), where therapeutic phlebotomy is routinely employed and requires careful titration to balance hematocrit control against the risk of inducing clinically significant iron restriction. In this setting, ZPP provides a direct functional readout of iron availability for erythropoiesis and thus enables monitoring of iron-restricted erythropoiesis before classical biochemical markers (e.g., ferritin) indicate manifest iron deficiency. This distinction is crucial, as early iron-restricted erythropoiesis may already contribute to symptom burden and altered erythropoietic dynamics while remaining undetected by standard laboratory parameters.

Accordingly, ZPP may serve as a valuable tool for guiding the frequency and intensity of phlebotomy in PV, allowing a more individualized approach that avoids excessive iron depletion while maintaining therapeutic efficacy. This concept is supported by prior work, particularly by Hastka et al., who demonstrated that ZPP reflects functional iron availability and is capable of detecting iron-deficient erythropoiesis at an early stage, preceding overt iron deficiency. Incorporating this perspective extends the clinical relevance of our findings beyond diagnostic refinement toward therapeutic monitoring and optimization in MPN.

ZPP was significantly higher in PV, MF, and MPN‑U than in ET, including many patients with normal or elevated ferritin, indicating functionally relevant iron deficiency that conventional parameters would miss. Stratification by driver mutation showed marked differences in ferritin and TSI between JAK2‑, CALR‑, MPL‑mutated and triple‑negative cases, but ZPP did not differ significantly across these groups, suggesting that iron‑restricted erythropoiesis is a shared downstream feature rather than a mutation‑specific phenomenon.

Functionally, ZPP behaved as a clinically meaningful marker. We observed a negative correlation between ZPP and Hb, and ZPP was elevated in three quarters of patients with CTCAE grade ≥ 2 anemia, linking higher ZPP directly to more severe anemia. In contrast, soluble transferrin receptor (sTFR) did not correlate convincingly with Hb, and its absolute levels were difficult to interpret against the background of clonal myeloproliferation and therapy, indicating that sTFR is at least partly uncoupled from classical ID physiology in MPN. ZPP and sTFR did correlate positively, and whenever sTFR suggested ID, ZPP was typically elevated, but ZPP also identified functional iron deficiency in patients whose ferritin, TSI, and sometimes sTFR would otherwise appear reassuring. The clinical implications are particularly clear in PV. Phlebotomy aims to induce iron‑deficient erythropoiesis to control erythrocytosis without tipping patients into overt, symptomatic iron deficiency. This balance is difficult to manage with ferritin and TSI alone, which are influenced by inflammation, recent intake, and treatment. ZPP allows a more precise distinction between controlled iron‑deficient erythropoiesis (moderately increased ZPP with preserved Hb) and manifest iron deficiency (markedly elevated ZPP with falling Hb), thereby informing the timing of transition from phlebotomy to cytoreductive therapy and guiding evaluation of fatigue and quality‑of‑life impairment in PV. Importantly, the diagnostic value of ZPP extends beyond PV and ET. In MF, both JAK2‑positive and non‑JAK2 patients displayed elevated ZPP despite high ferritin and often near‑normal TSI, indicating that iron‑restricted heme synthesis is a common hallmark of advanced disease, independent of driver mutation. Similarly, in MPN‑U and in ET patients with borderline or overlapping phenotypes, ZPP identified a subset of individuals who would be classified as iron‑replete based on ferritin and TSI but who are functionally iron deficient. This extends the concept from prior work that used TSI and JAK2 variant allele fraction for “diagnostic reassessment” in PV and ET by showing that ZPP adds a mechanistically grounded layer that can reclassify patients as functionally iron deficient even when storage and circulating markers are normal.

Overall, our findings underscore several limitations of traditional ID markers in MPN: ferritin is dominated by inflammation and disease stage, TSI loses discriminative power in advanced disease and ineffective erythropoiesis, and sTFR shows only limited correlation with anemia. In contrast, ZPP directly reflects iron incorporation into protoporphyrin IX and integrates iron supply, utilization, and erythroid activity into a single, relatively inflammation‑independent readout. Clinically, this supports the incorporation of ZPP into MPN diagnostic algorithms and trial designs to (i) detect iron‑restricted erythropoiesis despite “normal” ferritin/TSI, (ii) differentiate therapeutic from overt iron deficiency in PV, (iii) clarify the contribution of iron‑restricted heme synthesis to anemia in MF and MPN‑U, and (iv) provide a more robust biomarker for linking iron status to symptoms and quality of life [20] in prospective MPN studies.

## Data Availability

The datasets generated during and/or analyzed during the current study are potentially available from the corresponding author on reasonable request.
